# Evaluation of the postoperative analgesic effectiveness of erector spinae plane block ın patients undergoing robotic colorectal surgery: a prospective observational study

**DOI:** 10.1590/1806-9282.20260190

**Published:** 2026-07-24

**Authors:** Burcu İzgi Duman, Hürü Ceren Gökduman, Sercan Yüksel, Funda Gümüş Özcan

**Affiliations:** 1 Basaksehir Cam and Sakura City Hospital, Department of Anesthesiology – Istanbul, Turkey.; 2 Basaksehir Cam and Sakura City Hospital, Department of General Surgery – Istanbul, Turkey.

**Keywords:** Nerve block, Robotic surgical procedures, Colorectal surgery, Postoperative pain

## Abstract

**OBJECTIVE:**

As minimally invasive techniques, particularly robotic surgery, become more prevalent, effective postoperative pain management remains crucial in colorectal cancer surgery. The aim of this study was to evaluate the effect of an erector spinae plane block on postoperative pain management and opioid consumption in patients undergoing robotic colorectal surgery.

**METHODS:**

The study was designed as a prospective observational non-randomized study. After receiving ethical approval, 70 patients were evaluated prospectively: Thirty-six patients were in the erector spinae plane block group, and 34 patients were in the control group. Postoperative pain scores at rest and during deep breathing/coughing, as well as opioid consumption within the first 24 h, patient-controlled analgesia demand data, the need for rescue analgesics, postoperative nausea and vomiting, and complications were recorded.

**RESULTS:**

The erector spinae plane block group demonstrated significantly lower postoperative pain scores, reduced total opioid consumption, and reduced patient-controlled analgesia demand during the first 72 h compared with the control group. The erector spinae plane block group also had a longer time to the first analgesic request. The erector spinae plane block group also had a lower incidence of postoperative nausea and vomiting. Length of hospital stay and time to mobilization were similar between groups.

**CONCLUSION:**

Erector spinae plane block appears to be a safe and effective component of multimodal analgesia in robotic colorectal surgery. It was associated with lower postoperative pain scores, reduced opioid consumption, and fewer opioid-related side effects. Further multicenter randomized controlled studies are needed to confirm these findings.

## INTRODUCTION

Colorectal cancer is the third most commonly diagnosed malignancy worldwide, accounting for approximately 10% of all cancers^1^. Despite advances in chemotherapy, radiotherapy, and immunotherapy, surgical resection remains the cornerstone of treatment and the only potentially curative option for advanced-stage disease^2^. Minimally invasive techniques have replaced open surgery as the gold standard in colorectal cancer surgery due to reduced surgical trauma, faster recovery, improved cosmetic outcomes, and comparable long-term oncological results^3^.

Among minimally invasive approaches, robotic surgery has gained increasing popularity. Initially developed through collaboration between the US Army and National Aeronautics and Space Administration (NASA), robotic surgical systems offer advantages beyond conventional laparoscopy, including enhanced depth perception, tremor elimination, improved precision, three-dimensional visualization, and superior access to confined anatomical spaces such as the deep pelvis^4^. Today, it has become possible to manage it remotely via wired networks or 5G connections, and it is emerging as an area with increasing potential for development^5^. Meta-analyses have demonstrated that robotic surgery is associated with reduced blood loss, shorter hospital stay, and lower conversion rates compared with laparoscopic surgery, while providing comparable oncological outcomes^6^.

Postoperative pain remains a major clinical challenge and a significant contributor to opioid use worldwide. Inadequate pain control may lead to delayed recovery, prolonged hospitalization, increased pulmonary complications, and the development of persistent postoperative pain^7^. Although robotic surgery is generally associated with less postoperative pain than open approaches, effective analgesia remains essential.

Given the multifactorial nature of postoperative pain, multimodal analgesia is widely recommended, combining analgesic agents with different mechanisms of action and regional anesthesia techniques^8^. Epidural analgesia is no longer routinely recommended for minimally invasive colorectal surgery because of potential complications and the availability of less invasive alternatives^9^. Although the transversus abdominis plane block has been extensively investigated, its efficacy remains controversial due to inconsistent local anesthetic spread and uncertain superiority over trocar-site infiltration^10^.

The erector spinae plane (ESP) block is a relatively simple and safe regional analgesic technique that provides both somatic and visceral analgesia via paravertebral spread of local anesthetic. In this prospective study, we aimed to evaluate the effects of ESP block on postoperative analgesia and opioid consumption in patients undergoing robotic colorectal surgery.

## METHODS

This prospective, observational, non-randomized clinical study was conducted at the Department of Anesthesiology and Reanimation at the University of Health Sciences, Başakşehir Çam and Sakura City Hospital, after receiving approval from the Institutional Clinical Research Ethics Committee on October 11, 2023 (approval number E-96317027-574.10227004950). The study aimed to evaluate the effects of bilateral erector spinae plane block (ESPB) on postoperative analgesia, opioid consumption, and recovery outcomes in patients undergoing robotic colorectal surgery.

### Study population

Patients aged 18–70 years with American Society of Anesthesiologists (ASA) physical status I–III who underwent elective robotic colorectal surgery between November 2023 and November 2024 and provided written informed consent were included. Exclusion criteria comprised coagulation disorders, allergy to local anesthetics, impaired liver function, infection at the injection site, chronic analgesic use, body mass index (BMI) >35 kg/m^2^, ASA ≥IV, inability to cooperate with Numerical Rating Scale (NRS) assessment, and age outside the specified range.

All patients received standardized preoperative information regarding ESPB, patient-controlled analgesia (PCA), and NRS scoring. ESPB was performed preoperatively in patients who requested and consented to the procedure. Block performance, intraoperative management, and postoperative assessments were conducted by different anesthesiologists.

Randomization was not applied; volunteers were assigned to groups based on their request for preoperative ESPB.

### Erector spinae plane block procedure

Under standard ASA monitoring, patients received 2 mg intravenous midazolam for sedation. Ultrasound-guided bilateral ESPB was performed in the sitting position using a high-frequency (12–15 MHz) lineartransducer. After skin sterilization, the probe was placed longitudinally 2 cm lateral to the T11 spinous process. Pain transmission occurs via T10-L2 for the ascending and proximal transverse colon; and via T12-L2 for the distal transverse colon, descending, sigmoid colon, and upper rectum. Since it is known that the local anesthetic spreads craniocaudally after application to two dermatomes above and two dermatomes below the application level, T11 has been determined as the block level. The T11 level was selected to ensure adequate coverage of the relevant dermatomes involved in colorectal surgery, considering the expected craniocaudal spread of the local anesthetic. Following local infiltration with 1 mL of 2% lidocaine, a 22-gauge needle was advanced using an out-of-plane approach to contact the transverse process. Needle position was confirmed by hydrodissection, and 20 mL of 0.25% bupivacaine was injected into the erector spinae plane on each side under continuous ultrasound guidance. In all successful blocks, bilateral craniocaudal spread of the local anesthetic within the erector spinae plane was visualized under ultrasound guidance; sensory testing was not performed.

### Perioperative anesthesia management

General anesthesia was induced with intravenous propofol (2 mg/kg), fentanyl (2 µg/kg), and rocuronium (0.6 mg/kg), followed by orotracheal intubation. Anesthesia was maintained with sevoflurane to achieve a bispectral index of 40–60 and a remifentanil infusion (0.05–0.2 µg/kg/min). Rocuronium infusion was discontinued after robotic arm separation, and remifentanil was stopped approximately 10 min before the end of surgery. Prior to emergence, ondansetron (8 mg), paracetamol (1 g), and tramadol (1 mg/kg) were administered intravenously. Neuromuscular blockade was reversed with sugammadex (4 mg/kg), and patients were extubated.

### Postoperative follow-up

Patients were transferred to the post-anesthesia care unit (PACU) for monitoring of pain, vital signs, and adverse events. NRS scores and postoperative nausea and vomiting (PONV) were assessed at 0, 15, 30, and 60 min postoperatively. Patients with an NRS score ≥4 received 10 mg intravenous meperidine as rescue analgesia.

Postoperative analgesia was managed using a standardized multimodal protocol. All patients received intravenous paracetamol (1 g every 6 h). Opioid analgesia was provided using PCA with tramadol [(4 mg/mL) without a basal infusion, delivering 3-mL bolus doses with a 30-min lockout interval for 24 h]. Non-steroidal anti-inflammatory drugs (NSAIDs) or Cyclooxygenase (COX)-2 inhibitors were not routinely administered but could be used as rescue analgesics if clinically indicated. Antiemetic prophylaxis consisted of intraoperative ondansetron, with additional antiemetics administered post-operatively as needed. NRS scores and PONV were recorded at 2, 6, 12, 24, 48, and 72 h postoperatively. Total opioid consumption was obtained from PCA records. Time to first mobilization and length of hospital stay were documented.

### Outcomes

The primary outcome was total opioid consumption during the first 24 postoperative hours. Secondary outcomes included postoperative NRS scores, rescue analgesic requirements, incidence of PONV, time to mobilization, and length of hospital stay. All assessments were performed by anesthesiologists blinded to group allocation.

### Statistical analysis

The sample size calculation was based on detecting a 20% reduction in total tramadol consumption during the first 24 postoperative hours. Based on previously published studies and institutional experience, the mean tramadol consumption in the control group was assumed to be approximately 180 mg with a standard deviation of 70 mg. Detecting a 20% reduction (36 mg) with a power of 80% and a two-sided α level of 0.05 required a minimum sample size of 34 patients per group. Considering potential data loss or exclusions during the study period, we planned to include a total of 90 patients to ensure an adequate final sample size. Data normality was assessed using the Shapiro-Wilk test. Normally distributed variables were expressed as mean±standard deviation and compared using the Student’s t-test, while non-normally distributed variables were presented as median (25th–75th percentile) and analyzed using the Mann-Whitney U test. Categorical variables were expressed as frequency (percentage) and compared using the chi-square test. Postoperative pain scores were recorded at multiple time points up to 72 h. Given that the NRS represents ordinal data and repeated measurements did not satisfy the assumptions required for parametric repeated-measures analyses, a non-parametric approach was preferred. Changes in NRS scores over time within each group were analyzed using the Friedman test, and between-group comparisons at each postoperative time point were performed using the Mann-Whitney U test. Considering the sample size and study design, these methods were selected as conservative and robust approaches for within- and between-group comparisons. Statistical analyses were performed using Statistical Package for the Social Sciences version 21.0 (IBM Corp., Armonk, NY, USA) and MedCalc version 16.1 (MedCalc Software Ltd., Ostend, Belgium), with p<0.05 considered statistically significant.

## RESULTS

A total of 90 patients scheduled for robotic colorectal surgery at Başakşehir Çam and Sakura City Hospital were enrolled in this prospective observational study. 43 patients were included in the control group and 47 in the ESPB group. In both groups, three patients were excluded because of data loss from the PCA device, and six patients in each group were excluded due to incomplete postoperative follow-up data. In addition, in the ESPB group, two patients were excluded because the spread of the local anesthetic was considered inadequate on ultrasound examination, and the blocks were therefore regarded as unsuccessful.

**Figure 1:**
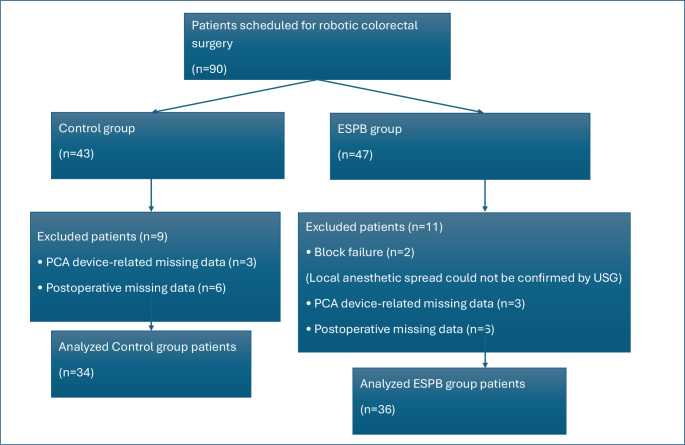
Mean and confidence interval for scores in question 4, according to observer group.

After these exclusions, 70 patients were included in the final analysis on a per-protocol basis (36 in the ESPB group and 34 in the control group; Figure 1).

Baseline demographic and clinical characteristics, including age, sex, BMI, American Society of Anesthesiologists (ASA) physical status, comorbidities, presence of Pfannenstiel incision, and anesthesia and surgical durations, were comparable between the groups (p>0.05) (Table 1).

**Table 1:** Demographic and operative characteristics of the groups.

		Control group n: 34		ESPB group n: 36		p
Age (years)	Mean±SD	58.06±10.32		58.17±11.84		0.968^*^
Gender	Male	22	64.71%	30	83.33%	0.075^+^
	Female	12	35.29%	6	16.67%	
BMI (kg/m^2^)	Mean±SD	26.01±4		27.97±5.05		0.077^*^
Comorbidity	No	15	44.12%	15	41.67%	0.836^+^
	Yes	19	55.88%	21	58.33%	
Diabetes mellitus	No	27	79.41%	29	80.56%	0.905^+^
	Yes	7	20.59%	7	19.44%	
Hypertension	No	23	67.65%	24	66.67%	0.930^+^
	Yes	11	32.35%	12	33.33%	
ASA risk score	I	1	2.94%	1	2.78%	0.782^+^
	II	26	76.47%	25	69.44%	
	III	7	20.59%	10	27.78%	
Pfannenstiel incision	No	15	44.12%	8	22.22%	0.090^+^
	Yes	19	55.88%	28	77.78%	
Duration of anesthesia (min)	Mean±SD	332.06±84.31		333.33±91.5		0.952^*^
Duration of surgery (min)	Mean±SD	277.65±87.18		283.33±93.9		0.794^*^

*Independent t-test.

^+^Chi-square test. ESPB: erector spinae plane block; SD: standard deviation; BMI: body mass index; ASA: American Society of Anesthesiologists.

Patients receiving ESPB demonstrated significantly reduced opioid requirements in the PACU compared with controls (median 0 [0–17.5] mg vs. 30 [0–40] mg, p=0.0001). Similarly, cumulative tramadol consumption via the PCA device during the first 24 h was significantly lower in the ESPB group (96 [84– 120] mg vs. 186 [120–240] mg, p=0.0001). Moreover, ESPB was associated with a prolonged time to first PCA demand (30 [20–60] vs. 0 [0–40] minutes, p=0.003) and fewer total PCA demands (9 [7–11.75] vs. 16 [10–20], p=0.0001) (Table 2).

**Table 2:** Postoperative total opioid consumption.

		Control group (n=34)	ESPB group (n=36)	p^†^
Opioid analgesic amount administered in PACU (mg)	Mean±SD	24.41±17.96	7.5±12.73	0.0001
	Median (IQR)	30 (0–40)	0 (0–17.5)	
Total tramadol consumption via PCA in the first 24 h (mg)	Mean±SD	176.12±66.31	106.14±29.35	0.0001
	Median (IQR)	186 (120–240)	96 (84–120)	
Time to first PCA demand (minutes)	Mean±SD	18.88±25.01	43.47±35.43	0.003
	Median (IQR)	0 (0–40)	30 (20–60)	
Total number of PCA demands	Mean±SD	14.97±5.51	9.44±2.6	0.0001
	Median (IQR)	16 (10–20)	9 (7–11.75)	

^†^Mann-Whitney U test. ESPB: erector spinae plane block; SD: standard deviation; PCA: patient-controlled analgesia; PACU: post-anesthesia care unit.

Mean resting NRS scores were significantly lower in the ESPB group at all assessed postoperative time points, including early PACU assessments and up to 72 h postoperatively (p=0.0001). Additionally, the ESPB group exhibited significantly lower PONV Impact Scale scores at 1 and 24 h post-operatively compared with the control group (p=0.009 and p=0.047, respectively). No significant differences were observed between the groups regarding time to first mobilization or length of hospital stay (p=0.869 and p=0.085, respectively).

Overall, these findings suggest that the use of erector spinae plane block in robotic colorectal surgery is associated with reduced postoperative opioid consumption and improved pain control, supporting a potential role for ESPB as a component of multimodal analgesia in minimally invasive colorectal surgery.

## DISCUSSION

In this study, the use of ESPB in patients undergoing robotic colorectal surgery was associated with significantly reduced postoperative opioid consumption, fewer PCA demands, longer time to first analgesic request, lower pain scores at rest and during deep breathing or coughing for up to 72 h, and reduced opioid requirements in the post-anesthesia care unit. In addition, the incidence of postoperative nausea and vomiting was lower in the ESPB group, while time to mobilization and length of hospital stay were comparable between groups.

The growing adoption of minimally invasive and robotic techniques has altered perioperative analgesic requirements, leading to reduced use of epidural anesthesia and increased interest in truncal blocks and other regional techniques^11^. In accordance with Enhanced Recovery After Current ERAS protocols address surgical processes with holistic approaches, comprehensively covering everything from maintaining the patient’s normothermia to good pain palliation and early initiation of oral feeding^12^. Multimodal analgesia combining regional techniques with non-opioid systemic agents is recommended to optimize pain control while minimizing opioid-related adverse effects^13^. In the present study, ESPB was incorporated into a standardized multimodal analgesic regimen alongside intravenous agents.

Previous studies evaluating pain management in robotic and laparoscopic surgery have demonstrated that ESPB is an effective and safe regional analgesic technique^14,15^. In laparoscopic colorectal surgery, randomized controlled trials have shown that ESPB significantly reduces postoperative opioid consumption and pain scores compared with control groups^16,17^. Our findings are consistent with these results; however, unlike some reports, we did not observe significant differences in hospital stay or time to mobilization. This discrepancy may be explained by the standardized early mobilization and discharge protocols routinely applied in our institution.

Systematic reviews and meta-analyses have further supported the efficacy of ESPB in minimally invasive abdominal surgery, demonstrating reductions in perioperative opioid use, postoperative pain scores, and postoperative nausea and vomiting^18,19^. In a randomized, double-blind study, Hou et al. reported that ESPB provided superior analgesia, improved quality of recovery, and higher patient satisfaction compared with the transversus abdominis plane block in laparoscopic colorectal surgery^20^. Although that study also showed shorter hospital stays in the ESPB group, such a difference was not observed in our cohort.

Current PROSPECT guidelines for laparoscopic colorectal surgery recommend a multimodal analgesic approach but do not specifically endorse truncal blocks because of insufficient evidence^8^. This limitation in the literature partly motivated the present study. By focusing specifically on robotic colorectal surgery and extending postoperative pain assessment up to 72 h, our findings add further evidence supporting the role of ESPB in this setting.

This study has several limitations that should be acknowledged. First, the observational design and the non-randomized allocation of patients to the ESPB or control group may introduce selection bias. Since ESPB was performed only in patients who requested and consented to the procedure, patients in the two groups may differ in ways that were not fully captured by baseline characteristics. Although demographic and clinical variables were comparable between groups, residual confounding cannot be completely excluded. Second, the success of the erector spinae plane block was not objectively confirmed using sensory testing; therefore, the possibility of incomplete or failed blocks cannot be entirely ruled out. Third, the study included a relatively small sample size and was conducted at a single center, which may limit the statistical power and generalizability of the findings. In addition, although non-parametric methods were used to analyze repeated pain measurements, more advanced statistical approaches such as mixed-effects models could provide a more robust evaluation of longitudinal data and account for within-subject variability. Furthermore, due to the retrospective design and limited sample size, no adjusted analyses (e.g., multivariable or propensity score methods) were performed; therefore, the results should be interpreted cautiously and considered primarily hypothesis-generating. Finally, although perioperative analgesic protocols were applied according to institutional practice, variations in additional analgesic requirements and individual pain perception may have introduced further confounding. For these reasons, the results should be interpreted with caution, and larger randomized controlled studies with standardized analgesic protocols and objective confirmation of block success are warranted to validate these findings.

## CONCLUSION

In this prospective observational study of patients undergoing robotic colorectal surgery, ESPB was associated with lower postoperative pain scores and reduced opioid consumption within the first 72 h after surgery. ESPB was also associated with a lower incidence of opioid-related adverse effects. The absence of differences in mobilization time and length of hospital stay may reflect the minimally invasive nature of robotic surgery and the standardized perioperative care applied to both groups. While these findings suggest that ESPB may be a useful component of multimodal analgesia in robotic colorectal surgery, larger multicenter randomized controlled trials are required to confirm these observations and to better define its role in standard postoperative analgesic protocols.

## Data Availability

The datasets generated and/or analyzed during the current study are available from the corresponding author upon reasonable request.

## References

[1] Sung H, Ferlay J, Siegel RL, Laversanne M, Soerjomataram I, Jemal A (2021). Global Cancer Statistics 2020: GLOBOCAN estimates of ıncidence and mortality worldwide for 36 cancers in 185 countries.. CA Cancer J Clin.

[2] Poston GJ, Figueras J, Giuliante F, Nuzzo G, Sobrero AF, Gigot JF (2008). Urgent need for a new staging system in advanced colorectal cancer.. J Clin Oncol.

[3] Butnari V, Sultana M, Mansuri A, Rao C, Kaul S, Boulton R (2024). Comparison of early surgical outcomes of robotic and laparoscopic colorectal cancer resection reported by a busy district general hospital in England.. Sci Rep.

[4] Gómez Ruiz M, Lainez Escribano M, Cagigas Fernández C, Cristobal Poch L, Santarrufina Martínez S (2020). Robotic surgery for colorectal cancer.. Ann Gastroenterol Surg.

[5] Artifon ELA, Kfouri F, Otoch JP, Kowalski LP, Nahas W, Ebaid G (2026). Feasibility of robotic telesurgery over wired and private 5 G networks within Brazil’s public health system (SUS): a pilot study.. Clinics (Sao Paulo).

[6] Zhang X, Wei Z, Bie M, Peng X, Chen C (2016). Robot-assisted versus laparoscopic-assisted surgery for colorectal cancer: a meta-analysis.. Surg Endosc.

[7] Hyland SJ, Brockhaus KK, Vincent WR, Spence NZ, Lucki MM, Howkins MJ (2021). Perioperative pain management and opioid stewardship: a practical guide.. Healthcare (Basel).

[8] Rosero EB, Joshi GP (2014). Preemptive, preventive, multimodal analgesia: what do they really mean?. Plast Reconstr Surg.

[9] Lirk P, Badaoui J, Stuempflen M, Hedayat M, Freys SM, Joshi GP (2024). PROcedure-SPECific postoperative pain management guideline for laparoscopic colorectal surgery: a systematic review with recommendations for postoperative pain management.. Eur J Anaesthesiol.

[10] Jones JH, Aldwinckle R (2020). Interfascial plane blocks and laparoscopic abdominal surgery: a narrative review.. Local Reg Anesth.

[11] Pirie K, Traer E, Finniss D, Myles PS, Riedel B (2022). Current approaches to acute postoperative pain management after major abdominal surgery: a narrative review and future directions.. BrJ Anaesth.

[12] Luo J, Zhou L, Lin S, Yan W, Huang L, Liang S (2020). Beneficial effect of fluid warming in elderly patients with bladder cancer undergoing Da Vinci robotic-assisted laparoscopic radical cystectomy.. Clinics (Sao Paulo).

[13] Nimmo SM, Foo ITH, Paterson HM (2017). Enhanced recovery after surgery: pain management.. J Surg Oncol.

[14] Durey B, Djerada Z, Boujibar F, Besnier E, Montagne F, Baste JM (2023). Erector spinae plane block versus paravertebral block after thoracic surgery for lung cancer: a propensity score study.. Cancers (Basel).

[15] La Via L, Cavaleri M, Terminella A, Sorbello M, Cusumano G (2024). Loco-regional anesthesia for pain management in robotic thoracic surgery.. J Clin Med.

[16] Park JW, Kim EK, Park S, Han WK, Lee J, Lee JH (2023). Erector spinae plane block in laparoscopic colorectal surgery for reducing opioid requirement and facilitating early ambulation: a double-blind, randomized trial.. Sci Rep.

[17] Kekul O, Ustun YB, Kaya C, Turunç E, Dost B, Bilgin S (2022). Analgesic efficacy of the bilateral erector spinae plane block for colorectal surgery: a randomized controlled trial.. J Anesth Analg Crit Care.

[18] Oraee S, Rajai Firouzabadi S, Mohammadi I, Alinejadfard M, Golsorkh H, Hatami S (2024). Erector spinae plane block for laparoscopic surgeries: a systematic review and meta-analysis.. BMC Anesthesiol.

[19] Sia CJY, Wee S, Au-Yong APS, Lie SA, Tan WJ, Foo FJ (2024). Analgesia efficacy of erector spinae plane block in laparoscopic abdominal surgeries: a systemic review and meta-analysis.. Int J Surg.

[20] Hou P, Liu W, Chen R, Mi H, Jia S, Lin J (2024). Comparison of erector spinae plane block and transverse abdominis plane block in postoperative recovery after laparoscopic colorectal surgery: a randomized, double-blind, controlled trial.. Perioper Med (Lond).

